# Bone Marrow Adipocytes in Multiple Myeloma: Emerging Immunometabolic Interactions and Implications for Immunotherapy

**DOI:** 10.3390/cancers18193213

**Published:** 2026-10-05

**Authors:** Adriana Roque, Artur Paiva, Raquel Seiça, Catarina Geraldes

**Affiliations:** 1 Clinical Hematology Unit, Unidade Local de Saúde de Coimbra (ULS Coimbra), 3004-561 Coimbra, Portugal; 2 Institute of Physiology, Faculty of Medicine, University of Coimbra (FMUC), 3000-548 Coimbra, Portugal; 3 University Clinics of Hematology and Oncology and Laboratory of Oncobiology and Hematology, Faculty of Medicine, University of Coimbra, 3000-548 Coimbra, Portugal; 4 Unidade Funcional de Citometria de Fluxo, ULS Coimbra, 3004-561 Coimbra, Portugal; 5 Faculty of Medicine, Coimbra Institute for Clinical and Biomedical Research (iCBR), University of Coimbra, 3000-548 Coimbra, Portugal; 6 Ciências Biomédicas Laboratoriais, ESTESC-Coimbra Health School, Instituto Politécnico de Coimbra, 3045-043 Coimbra, Portugal; 7 Group of Environmental Genetics of Oncobiology (CIMAGO), Faculty of Medicine, University of Coimbra (FMUC), 3004-504 Coimbra, Portugal; 8 Center for Innovative Biomedicine and Biotechnology (CIBB), University of Coimbra, 3004-504 Coimbra, Portugal; 9 Clinical Academic Center of Coimbra (CACC), 3000-076 Coimbra, Portugal

**Keywords:** multiple myeloma, bone marrow adipocytes, immunometabolism, lipid metabolism, bone marrow microenvironment, T-cell dysfunction, natural killer cells, monoclonal antibodies, bispecific antibodies, CAR T cell therapy

## Abstract

Immunotherapy has revolutionized the treatment of multiple myeloma (MM), but durable immune control remains limited, increasing interest in how the bone marrow (BM) microenvironment influences antitumor immunity. BM adipocytes (BMAds) are increasingly recognized as metabolically active components of the BM. This review summarizes the current evidence linking BMAds to myeloma cell survival and immune cell function, particularly T cell activity. It also discusses their potential relevance to monoclonal antibodies, bispecific antibodies, and chimeric antigen receptor (CAR) T-cell therapy. Preclinical studies have shown that myeloma-associated BMAds undergo lipid depletion and acquire inflammatory and senescence-associated secretory features. BM lipid exposure is associated with impaired CD8^+^ T-cell metabolic fitness, whereas leptin receptor activation suppresses invariant natural killer T-cell anti-myeloma activity. Evidence concerning conventional natural killer cells and other immune compartments remains limited. Available clinical evidence is predominantly cross-sectional and therefore does not establish a direct role for BMAds in resistance to current immunotherapies or clarify whether they influence therapeutic target availability or function as clinically relevant bystander targets. Patient- and treatment-related factors, including aging and obesity, may further reshape the adipocytic-stromal and immune landscape of the marrow, potentially modifying local responses to immunotherapy. Future studies combining patient-derived multicellular models with spatial and longitudinal approaches should determine how BMAd-associated metabolic, inflammatory, and adipokine signaling affect immune control and treatment outcomes in MM and whether these processes can be therapeutically modulated.

## Simple Summary

Immunotherapy has transformed the treatment of multiple myeloma (MM). However, many responses are not durable, which may partly reflect the bone marrow microenvironment, where myeloma cells interact with immune cells, stromal cells, and bone marrow adipocytes (BMAds). BMAds are metabolically active cells that release lipids and soluble mediators and engage in bidirectional crosstalk with myeloma cells. This review explores how BMAds support myeloma cell survival and may influence immune cell function, particularly T cells, with limited evidence regarding natural killer (NK) cells. We also discuss the potential relevance of these relationships for monoclonal antibodies, bispecific antibodies, and chimeric antigen receptor (CAR) T-cell therapy. Current evidence links BMAds to immunometabolic remodeling of the myeloma niche but does not demonstrate that they directly cause immunotherapy resistance. Future studies should assess the therapeutic potential of modulating BMAd-associated lipid metabolism, inflammatory signaling, and adipokine activity to improve immune control in patients with MM.

## 1. Introduction

Multiple myeloma (MM) is a hematologic malignancy characterized by clonal expansion of malignant plasma cells (PC) and profound immune dysregulation [1,2]. MM develops mainly within the bone marrow (BM), where the stromal, immune, vascular, bone, and adipocytic compartments influence tumor cell survival, immune escape, and treatment response [2]. This multicellular context is particularly relevant to current immunotherapeutic approaches, including monoclonal antibodies (mAbs), antibody-drug conjugates (ADCs), bispecific antibodies (BsAbs), and chimeric antigen receptor (CAR) T-cell therapy, whose efficacy depends not only on tumor antigen expression but also on the functional competence of endogenous or engineered immune cell effectors [2,3,4].

Preliminary transcriptomic profiling of non-hematopoietic BM cells suggests that stromal remodeling is detectable across the monoclonal gammopathy of undetermined significance (MGUS)—smoldering MM (SMM)—MM continuum [5]. Whole BM deconvolution has also indicated a stage-associated shift in the immune cell composition from MGUS to MM, although these cell estimates were computationally derived [6]. In newly diagnosed MM (NDMM), single-cell profiling further identifies distinct immune states associated with rapid progression and survival [7]. In NDMM, transcriptomic deconvolution of whole-BM biopsies indicated that the non-tumor microenvironment adds prognostic information beyond tumor burden and established clinical and tumor risk variables, supporting the prognostic relevance of BM microenvironment remodeling [6].

Bone marrow adipocytes (BMAds) have historically been underappreciated among the cellular constituents of the BM niche. BMAds were long viewed as passive space-filling cells but are now recognized as metabolically active secretory cells capable of influencing both tumor-cell metabolism and the local immune environment [8,9].

BM adiposity increases with age and is particularly relevant to MM, which predominantly affects older adults [1,10,11,12]. In addition to their abundance in the aging BM, BMAds appear to be functionally integrated into the myeloma niche.

Preclinical studies suggest that adipocytes may support MM cell growth and survival, promote metabolic adaptation, and contribute to resistance to conventional therapies [8,13,14,15,16]. BMAd-associated lipids, adipokines, and inflammatory signals can affect immune-cell biology and may therefore be relevant to mAbs, BsAbs, ADCs, and CAR T-cell therapy. Direct effects of BMAds on immunotherapy efficacy, however, have not been demonstrated.

This review evaluates the evidence linking BMAds to immunometabolic remodeling of the myeloma BM niche, with particular emphasis on immune cell fitness and potential implications for immunotherapy. We included findings from non-MM marrow or adipose-tissue models that provide relevant biological context, although their applicability to MM remains to be established.

## 2. Bone Marrow Adipocytes and Immune Regulation in Multiple Myeloma

### 2.1. Bone Marrow Adipocyte Biology and Remodeling in Multiple Myeloma

BMAds arise from bone marrow stromal cells (BMSCs) that also give rise to osteoblast-lineage cells, making the adipocyte-osteoblast balance a key determinant of BM niche composition [17,18,19]. This balance is particularly relevant in MM, where defective osteoblastogenesis, altered bone marrow adipose tissue (BMAT) composition and function, and stromal remodeling coexist within the same pathological niche [20,21].

Adipogenic remodeling in MM is not merely a passive consequence of adipocyte-mediated replacement of the hematopoietic marrow. Co-culture studies have indicated that myeloma cells can reprogram BMSCs and preadipocytes, suppressing osteogenic differentiation while inducing adipogenic and lipid-handling transcriptional programs [22,23]. In BMSCs, the shift from osteogenic to adipogenic differentiation is linked to peroxisome proliferator-activated receptor gamma (PPARγ)-related signaling [20]. This adipogenic shift may also be reinforced by sclerostin-mediated inhibition of *Wnt* signaling, linking myeloma bone disease to stromal adipogenic remodeling. Although early studies reported sclerostin secretion by MM cells, subsequent studies indicated that osteocytes and other osteolineage cells may represent more important local sources in myeloma bone disease. Sclerostin suppresses osteoblast differentiation and promotes adipogenesis in BMSCs, but its immune consequences remain unclear [24,25,26].

Mechanosensitive pathways may also contribute to the regulation of BMAT. In non-myeloma models, *Piezo1* signaling in BMSCs has been implicated in the control of adipogenic differentiation and marrow adiposity, although its relevance to MM remains unclear [27]. Developmental studies have further refined this lineage model by identifying C-X-C motif chemokine ligand 12 (CXCL12)-expressing adipogenic stromal subsets and bone-specific adipogenic programs that may give rise to BMAds with distinct lipid storage and endocrine properties [28,29]. This cellular and lineage heterogeneity argues against treating adipocyte-lineage cells as a single functional compartment [17,30].

In the 5TGM1 murine model, single-cell RNA sequencing (scRNA-seq) of enriched non-hematopoietic BMSCs following myeloma engraftment revealed inflammatory and oxidative stress programs, along with a shift in stromal differentiation trajectories away from osteocyte-associated states and toward adipocyte lineage states [31]. In human MM marrow, small adipocytes expressing insulin-like growth factor-binding protein 2 (IGFBP2), some of which co-express adiponectin, have been identified [32]. This adipocyte population is less abundant in high-risk than in low-risk MM, and its co-culture with myeloma cells decreases IGFBP2 and adiponectin expression in normal BMSCs [32]. These observations suggest that the loss of the IGFBP2^+^/adiponectin^+^ adipocyte lineage is a feature of BMAT remodeling in MM.

BMAds are heterogeneous and biologically distinct from peripheral adipocytes, with distinct lipid, metabolic, secretory, and inflammatory characteristics [33,34]. Proteomic and lipidomic profiling of primary human BMAds indicates that they have limited baseline lipolytic activity, are enriched in cholesterol metabolism proteins, and accumulate monoacylglycerol, features that are not fully recapitulated by BMSC-derived adipocytes [33]. Femoral BMAds are smaller, store less triglyceride, express lower CD36 levels, and secrete higher levels of pro-inflammatory cytokines than subcutaneous adipocytes [34]. BMAd heterogeneity also varies according to anatomical location. BMAT can be broadly divided into regulated BMAT (rBMAT), which predominates in proximal hematopoietically active skeletal sites and is characterized by individual adipocytes interspersed among hematopoietic cells, and constitutive BMAT (cBMAT), which is found more distally, contains larger adipocytes, appears earlier in development, and is relatively resistant to systemic perturbations [17]. However, the rBMAT/cBMAT distinction was established largely from region-specific anatomical and functional studies in rodents and has not yet been validated as representing functionally distinct compartments in human MM marrow [17,35]. This distinction is particularly relevant in MM, as myeloma infiltration and bone disease preferentially involve red marrow-rich skeletal regions, namely the axial skeleton and proximal long bones. Therefore, adipocytes and adipocyte-lineage progenitors within rBMAT-like areas may be preferentially exposed to tumor-derived inflammatory and metabolic signals, although this remains a spatially untested hypothesis in human myeloma BM [17,36,37]. Consequently, any distinction between rBMAT and cBMAT in myeloma-associated immune dysfunction should be regarded as a testable hypothesis, rather than an established compartment-specific mechanism.

Studies using 3T3-L1 preadipocytes and mature adipocytes have shown distinct effects on MM cells. Preadipocyte co-culture enhanced MM cell migration in vitro, and MM cells previously exposed to preadipocytes exhibited earlier and greater tumor growth in the bone following intravenous inoculation. In contrast, mature adipocytes activate extracellular signal-regulated kinase (ERK) signaling in MM cells, and mature adipocyte-conditioned medium increases MM cell growth in vitro [38]. These results underscore that preadipocytes, in vitro BMSC-derived adipocytes, and mature primary BMAds are not interchangeable models and that observations from each system should be interpreted in the context of the adipocyte lineage state and anatomical BMAT compartment represented [17,33,39].

Mature BMAds are large, buoyant, and fragile, complicating their isolation and limiting their analysis by conventional flow cytometry. Current BMAS recommendations describe specific procedures for BMAT and BMAd isolation, storage, and characterization, including histological, imaging, molecular, and omics-based approaches, while emphasizing the need for greater methodological standardization [40]. For routine tissue-based assessments, histological and imaging-based approaches can provide relatively accessible information on BMAT abundance and adipocyte morphology, whereas functional studies require dedicated procedures to isolate and maintain viable mature BMAds [40]. These limitations are particularly relevant when studying BMAd-immune interactions, which require viable primary adipocytes to be incorporated into multicellular systems with MM cells and immune effectors.

These distinctions are also relevant for immune regulation. Differences in adipocyte lineage state, maturation, and anatomical location may alter the amount and composition of lipids, adipokines, and inflammatory mediators released into the marrow niche [17,33,34]. Because primary human BMAds differ from subcutaneous adipocytes and conventional BMSC-derived adipocytes in their metabolic, secretory, and immune regulatory properties [32,33], the immune effects observed in one model should not be directly generalized to another. In MM, the strongest available links are lipid-associated CD8^+^ T-cell dysfunction and leptin-mediated impairment of invariant natural killer T (iNKT) cell anti-myeloma activity [41,42]. However, these effects have not been compared across adipocyte lineage states or between different BMAT compartments.

In co-culture systems, MM cells induce lipolysis in adipocytes, as reflected by reduced lipid droplet size and increased glycerol secretion [16]. The free fatty acids (FFA) released during this process are taken up by MM cells through fatty acid transport proteins 1 and 4 (FATP1 and FATP4), which are upregulated in MM cells [16]. Consistent with FATP-mediated FFA uptake, pharmacological blockade of FATPs with lipofermata reduced lipid uptake by MM cells in a co-culture model [16].

Beyond lipid remodeling, myeloma-exposed BMAds acquire a senescence-associated secretory phenotype (SASP), with increased senescence-associated β-galactosidase activity and increased expression of inflammatory and stress-related mediators, including IL-6, CXCL1, and CXCL2, along with increased expression of senescence-associated markers in the BM of tumor-bearing mice [43].

In vitro, conditioned medium from myeloma-modified or experimentally senescent adipocytes reduced dexamethasone-induced cell cycle arrest and apoptosis in dexamethasone-sensitive MM.1S cells. Treatment of adipocytes with the senolytic combination of dasatinib and quercetin, agents that selectively eliminate senescent cells, attenuated this protective effect [43]. However, these findings were obtained using a cell line model and were not validated in primary cells from patients with relapsed/refractory MM (RRMM).

Mature adipocytes can promote autophagy in MM cells, as reflected by increased levels of autophagy-related protein 3 (ATG3), autophagy-related protein 5 (ATG5), and microtubule-associated protein 1 light chain 3 (LC3)-I/II, following exposure to adipocyte-conditioned medium. Adipocyte-conditioned medium also reduced chemotherapy-induced apoptosis, an effect partly mediated by adipocyte-derived leptin and adipsin [15]. Adipocyte-mediated chemoprotection may also involve direct drug sequestration, as doxorubicin was shown to accumulate within adipocyte lipid droplets in a three-dimensional BMAd model, reducing its cytotoxic activity against MM cells [44].

Preliminary data suggest reciprocal signaling between BMAds and MM cells. BM adipocyte-conditioned medium increased MM.1S cell numbers, whereas transwell co-culture induced transcriptional signatures related to signal transducer and activator of transcription 3 (STAT3), leptin, and interleukin-6 (IL-6) signaling, along with increased expression of survival-associated genes in MM cells. Conversely, exposure to MM cells induces lipid metabolism genes in BMAds [45].

These findings, together with the SASP alterations observed in myeloma-exposed BMAds, suggest that changes in BMAd secretory and metabolic activity, rather than adipocyte abundance alone, may contribute to MM cell survival within the BM niche [15,43].

### 2.2. Spatial and Longitudinal Context of BMAd-Immune Interactions

Evidence for early BMAT remodeling before overt MM is limited. In a retrospective, artificial intelligence (AI)-assisted morphometric analysis of unstained MGUS biopsies, patients who subsequently progressed to MM had lower BMAd density and a shift toward larger and rounder adipocytes, despite no difference in the total BMAT fraction [46]. These findings suggest that BMAd morphology may be a progression-associated biomarker. However, they did not demonstrate early expansion of total BMAT or establish a uniform longitudinal trajectory from MGUS or SMM to MM. In contrast, a cohort study focusing on BM trephine biopsies from patients with established MM reported significant BMAT loss with myeloma cell infiltration and BMAT restoration after MM treatment [43]. These findings align with local adipocyte delipidation in established disease and co-culture evidence showing that MM cells induce adipocyte lipid depletion [16].

This dynamic remodeling is relevant to the immune niche because lipid-depleted tumor-adjacent adipocytes may release metabolites and inflammatory mediators that modulate the fitness of local immune cells in the tumor microenvironment.

Comparison of non-adipocytic cells recovered from isolated BMAT with cells from the remaining aspirate showed enrichment of clonal MM cells in the BMAT-associated fraction in 32 of 34 samples. Within this fraction, recovered MM cells showed higher expression of fatty acid-binding protein 5 (FABP5), whereas non-adipocytic cells displayed increased oxygen consumption and extracellular acidification rates compared to adipocytes. In contrast, the increase in natural killer (NK) cell frequency observed in aspirates from MGUS, NDMM, and follow-up samples relative to non-tumor controls was not observed in the corresponding BMAT fractions, which was particularly evident for cytotoxic CD16^high^CD56^low^ NK cells [47]. These preliminary findings suggest that BMAT may represent a distinct plasma cell-supportive marrow subcompartment with an immune composition that differs from that of the surrounding aspirate. However, cross-sectional data do not establish immune exclusion or direct impairment of NK cell proliferation or function.

Non-invasive MRI-based approaches may help monitor changes in marrow fat over time, although their interpretation is limited by MRI-derived metrics that are not directly interchangeable across available studies. In NDMM, the lesion-level signal fat fraction (sFF), measured using a modified Dixon MRI sequence, increased significantly after two cycles of bortezomib-based induction in patients who achieved a response, whereas no significant change was observed in non-responders [48]. In contrast, the vertebral fat fraction (FF) and chemical-shift-encoded proton-density fat fraction (CSE-PDFF), which provide quantitative estimates of vertebral BM fat, have only been assessed in preliminary studies to date [49,50].

Current data indicate that BMAT remodeling in MM is spatially heterogeneous and context-dependent, but its longitudinal trajectory remains unknown. Patient-matched longitudinal studies integrating marrow imaging and spatial tissue profiling are needed to clarify whether BMAT remodeling and adipocyte-to-MM lipid transfer change with disease progression or treatment response [16,47].

## 3. Adipocyte-Derived Mediators in Multiple Myeloma Immunity

BMAd remodeling may influence the myeloma niche through soluble and metabolic signals, affecting tumor cell behavior and local immune regulation. Table 1 summarizes BMAd-associated mechanisms with direct or potential immunological relevance, including BM lipid availability, adipokine signaling, inflammatory remodeling, and MM cell survival.

### 3.1. Adipokines, Soluble Mediators, and Immune Surveillance

Among the adipokines implicated in MM, leptin and adiponectin have opposing roles. Leptin promotes MM cell survival and can impair anti-myeloma immune responses, whereas adiponectin exerts tumor-suppressive effects in preclinical MM models, highlighting the contrasting regulatory effects of adipokines within the BM microenvironment [42,51,53].

Higher plasma leptin concentrations have been reported in patients with NDMM, although epidemiological studies have not consistently linked circulating leptin levels to the risk of MM [56,57].

In vitro, leptin increases AKT and STAT3 phosphorylation in MM cells, promoting their proliferation [51]. In co-cultures with mature adipocytes, MM cells showed reduced bortezomib-induced apoptosis, whereas leptin receptor (LEPR) blockade attenuated the adipocyte-associated proliferation [51]. Leptin and MM cells synergistically impair iNKT-cell anti-myeloma activity through LEPR signaling, whereas LEPR blockade enhances iNKT-cell-mediated antitumor activity [42].

In BMAd–myeloma co-culture models, myeloma cells downregulate adiponectin expression in BMAds, at least partly through TNF-α [52]. Host-derived adiponectin also suppresses MM growth and myeloma-associated bone disease in preclinical models [53]. These data are consistent with a tumor-suppressive role for adiponectin in MM, and its downregulation may reduce protective signaling from the adipocyte compartment. The immune effects of adiponectin are less clear and are derived mainly from non-MM models. Adiponectin has been shown to reduce NK-cell cytotoxicity [58] and to impair dendritic cell-mediated antitumor T-cell immunity [59]. In non-MM settings, human femoral BMAds reduce immunoglobulin G secretion by plasma cells [34], whereas adiponectin deficiency in obese mice is associated with BM inflammation and impaired antibacterial activity of hematopoietic cells [60]. However, these findings do not establish a direct link between myeloma-associated adiponectin loss and impaired anti-myeloma immunity, immunoparesis, or infection susceptibility in patients with MM.

Other adipokines illustrate the tumor-supportive potential of adipocyte-derived signaling. Resistin protects MM cells from apoptosis through nuclear factor kappa B (NF-κB) and phosphoinositide 3-kinase (PI3K)/AKT signaling and promotes adenosine triphosphate (ATP)-binding cassette (ABC) transporter-mediated multidrug resistance through promoter demethylation [61], whereas adipsin has been implicated in Janus-associated kinase (JAK)/STAT3-dependent activation of autophagy [15].

Patients with MM exhibited higher serum chemerin levels than healthy controls, with the highest levels observed in those with International Staging System (ISS) stage III disease [62]. Chemerin is expressed by BMSCs from patients with MM and secreted by preadipocytes generated in vitro from healthy donor BMSCs [62]. In primary MM samples, C-C chemokine receptor-like 2 (CCRL2) mRNA was broadly expressed, whereas the expression of chemerin chemokine-like receptor 1 (CMKLR1), a signaling chemerin receptor, was detected only in a subset of samples. This receptor expression pattern suggests that chemerin responsiveness may be restricted to a subset of MM cells [62]. However, this study did not assess chemerin secretion by mature primary human BMAds or its functional effects on MM cells [62].

A small cross-sectional case–control study reported higher circulating resistin, chemerin, and adipsin levels in patients with MM than in healthy controls. Resistin levels were lower in patients with R-ISS stage II–III disease than in those with R-ISS stage I, whereas adiponectin levels were lower in patients with progressive disease than in those in complete remission. Given the cross-sectional design, these findings indicate an association with disease stage and response status rather than an established prognostic value [63].

In addition to classical adipokines, adipocyte-derived soluble factors may also alter the transcriptional programs in MM cells. In BMAd-MM models, these factors suppressed canonical aryl hydrocarbon receptor (AhR) signaling in human MM cell lines, whereas lower expression of AhR target genes was associated with poorer overall survival in a public MM dataset [64]. The findings indicate soluble crosstalk between adipocyte-like cells and MM cells in this model, but primary human BMAds and immune-cell responses were not assessed.

The local sources of these adipokines within the MM marrow remain incompletely defined, and direct immune effects are established only for selected pathways, most clearly the leptin-iNKT axis.

### 3.2. Lipid Transfer and Immune Metabolic Dysfunction

The lipolysis-FATP1/4 axis described previously provides a mechanistic basis for adipocyte-to-MM lipid transfer, but immune-cell endpoints were not assessed in that study [16]. FABP4 and FABP5 may provide an additional link between lipid metabolism, the BM microenvironment, and MM progression. In a mouse model of MM using global *Fabp4/Fabp5* double-knockout mice, MM engraftment and tumor incidence were reduced, with improved survival under high-fat diet conditions. However, the systemic nature of *Fabp4/Fabp5* knockout precluded the identification of the specific microenvironmental cell population responsible for these effects [65]. In patient-derived MM data, lower FABP5 expression in granulocyte-monocyte progenitors was associated with improved survival. This association raises the possibility of a myeloid contribution but does not establish a causal role for FABP5 in myeloid immunosuppression [65]. FABP5 also exerts direct effects on MM cells. In MM cell models, pharmacological inhibition of FABPs reduces proliferation, increases apoptosis, and impairs mitochondrial respiration. Genetic loss of *FABP5* also reduced MM cell proliferation and increased apoptosis. However, the variable activity of pharmacological FABP inhibition across mouse models limits the conclusions regarding its translational potential [66].

In adipocyte-MM co-cultures, the effects of FFAs on MM cells were concentration-dependent: lower concentrations promoted MM cell proliferation, whereas higher concentrations induced ferroptosis-associated lipotoxicity [16]. The contribution of specific FFAs, including arachidonic acid, and the relevance of these findings to physiological lipid exposure within the BM niche remain unclear.

Among the immune cell populations, CD8+ T cells provide the clearest evidence that lipid exposure in the MM marrow impairs metabolic fitness and effector function. In MM, CD8^+^ T cells exhibit reduced function and mitochondrial mass, along with increased uptake of long-chain FFAs [41]. In ex vivo assays, reducing extracellular lipids or inhibiting fatty acid transport protein 1 (FATP1) improved CD8^+^ T-cell metabolic fitness and effector function. However, the cellular source of relevant BM lipids has not yet been identified [41]. The extent to which BMAd-derived FFAs simultaneously support MM cell metabolism and compromise CD8^+^ T cell function in vivo has not been directly tested [16,41].

### 3.3. Senescence-Associated and Epigenetic Remodeling of the Myeloma Niche

Senescence-associated remodeling may alter the inflammatory and metabolic properties of the myeloma niche. However, the direct effects of SASP factors on specific immune cell populations in MM remain unclear [43].

In 3T3-L1 models, conditioned media from both preadipocytes and mature adipocytes promoted MM cell chemotaxis [38]. In vitro, neutralization of monocyte chemotactic protein 1 (MCP-1) or C-X-C motif chemokine ligand 12/stromal cell-derived factor 1 alpha (CXCL12/SDF-1α) reduced this response [38]. In another study, myeloma cell-derived mitochondrial DNA was reported to increase inflammatory signatures, including IL-6 expression, in BM-derived adipocytes. Blocking toll-like receptor 9 (TLR9) attenuated IL-6 induction. MM cells co-cultured with mtDNA-activated adipocytes showed increased FFA uptake and β-oxidation-related metabolism [67].

Adipocyte-derived exosomes may also contribute to MM cell chemoresistance. In preclinical models, exosomes from MM-associated adipocytes protected MM cells from chemotherapy-induced apoptosis through the transfer of pro-survival long non-coding RNAs, a process modulated by EZH2-dependent mechanisms [55].

Epigenetic reprogramming can lead to persistent niche dysfunction. In adipocytes exposed to myeloma cells, integrin α6 expressed by myeloma cells activated ERK1/2 and NF-κB signaling. This activation increased enhancer of zeste homolog 2 (EZH2) expression and enriched histone H3 lysine 27 trimethylation (H3K27me3) at the PPARγ promoter region. This epigenetic program alters adipokine expression, and the conditioned medium from these adipocytes promotes osteoclastogenesis and impairs osteoblast differentiation. Adipocyte-specific EZH2 deletion attenuated these bone changes in mouse models [21]. Although these mechanisms were primarily defined in relation to tumor-supportive effects and bone remodeling, they may indirectly shape myeloma immunity by sustaining an inflammatory, metabolically altered niche with persistent adipokine signaling.

For extracellular vesicles and epigenetic transfer mechanisms, the immune consequences are still inferred mainly from tumor survival, chemoresistance, and bone remodeling readouts.

## 4. Immune Dysfunction in Adipocyte-Rich Marrow Niches

**Figure 1:**
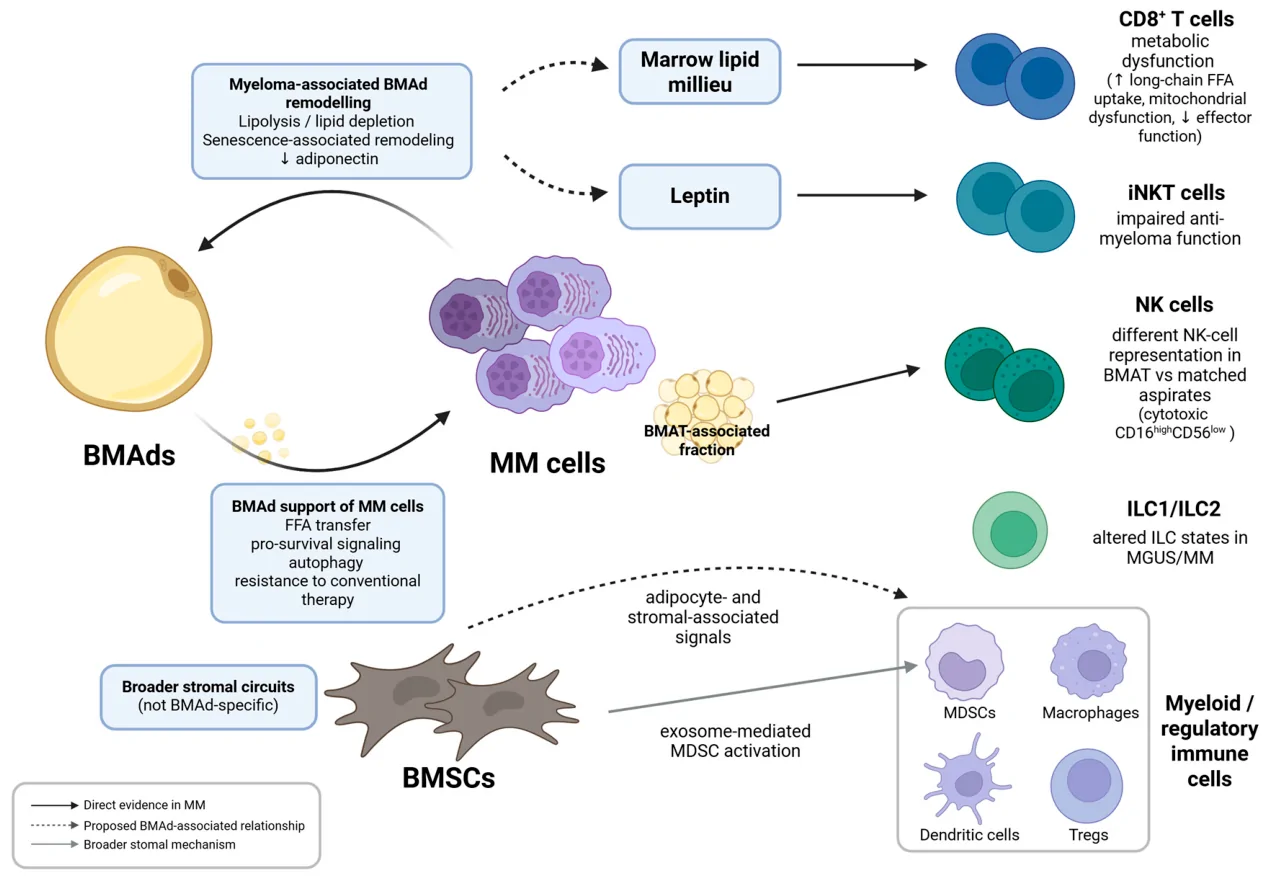
BMAd-centered immunometabolic interactions in the MM marrow niche. Myeloma-driven remodeling of BMAds may support malignant PC survival and shape local immune cell fitness through lipid-related, adipokine-associated, and inflammatory niche signaling. The figure integrates BMAd-myeloma interactions with immune and stromal compartments relevant to the myeloma BM microenvironment. BMAd, bone marrow adipocyte; BMAT, bone marrow adipose tissue; BMSC, bone marrow stromal cell; FFA, free fatty acid; iNKT, invariant natural killer T cell; ILC, innate lymphoid cell; MDSC, myeloid-derived suppressor cell; MM, multiple myeloma; NK, natural killer; Treg, regulatory T cell. Created with https://BioRender.com/u3ziab1 on 19 August 2026.

The strength of evidence for BMAd-immune interactions varies substantially among different immune cell populations. Figure 1 summarizes the BMAd-MM crosstalk and its potential links with immune cell dysfunction within the MM BM niche.

### 4.1. T-Cell Dysfunction in the Myeloma Bone Marrow Niche

BM lipid exposure is associated with impaired CD8^+^ T-cell metabolic fitness and function, although the contribution of BMAds to this lipid pool remains unresolved [41].

BMSCs from patients with MM (MM-BMSCs) can suppress CD8^+^ T-cell cytotoxicity via programmed cell death protein 1 (PD-1)/programmed death ligand 1 (PD-L1) signaling, thereby promoting CD8^+^ T-cell apoptosis and reducing perforin and granzyme B release [68]. MM-BMSCs can also inhibit CD4^+^ T-cell proliferation, promote T-cell senescence, and drive T helper 17 (Th17) differentiation through FAPα-associated phosphoinositide 3-kinase (PI3K) and transforming growth factor beta (TGF-β) signaling pathways [69]. MM-BMSCs also produce higher IL-6 and lower interleukin-10 (IL-10) than healthy donor BMSCs, increasing the Th17/Treg ratio during T cell co-culture experiments [70]. In adipocyte-rich marrow, these stromal checkpoint and cytokine signals may converge with BMAd-derived lipids and inflammatory programs to restrict local T-cell fitness.

This distinction is clinically relevant because MM-associated T-cell dysfunction can include senescence-like states that are not captured by a uniform PD-1^high^ exhaustion model. Clonally expanded T cells in MM display a KLRG-1^+^CD57^+^CD160^+^CD28^−^ senescent effector phenotype with relatively low expression of PD-1 and cytotoxic T lymphocyte-associated protein 4 (CTLA-4). Induction therapy and autologous stem cell transplantation (ASCT) further deplete *naïve* T cell populations and remodel T cell metabolism [71,72].

Single-cell studies have further identified stage- and outcome-associated immune remodeling. In a study reporting a large MGUS-SMM-NDMM single-cell dataset, T cell receptor repertoire diversity was lower in SMM and NDMM than in MGUS and was further reduced in patients with SMM who subsequently progressed to MM [73]. Beyond T-cell repertoire changes, single-cell profiling in NDMM has identified a proinflammatory immune SASP associated with rapid clinical progression [7].

### 4.2. NK Cells, iNKT Cells, Innate Lymphoid Cells, and Regulatory T-Cell Response

The most direct evidence linking BMAds to innate lymphocyte dysfunction in MM involves leptin-mediated inhibition of iNKT cell anti-myeloma activity. Leptin receptor blockade restored iNKT-cell activity in preclinical models [42]. Evidence involving conventional NK cells is more limited. In paired samples from patients with MM, differences in cytotoxic NK cell representation were observed between BMAT-associated fractions and BM aspirates, although NK cell function was not assessed [47].

Other components of the BM stroma can also impair NK-cell function. MM-derived BMSCs reduce NK cell cytotoxicity and can induce NK cell dysfunction through CD155/TIGIT signaling [74,75]. These findings are relevant to BMSCs rather than mature BMAds. The relevance of BMAds to NK cell function and NK cell-dependent activity of anti-myeloma mAbs has not been directly examined.

Alterations in ILC and Treg populations have been described in MGUS and MM [76,77,78], but a direct relationship with BMAds has not been demonstrated.

### 4.3. Myeloid Cells and Dendritic Cells

Direct evidence linking mature BMAds to myeloid or dendritic-cell (DC) dysfunction in MM is limited. MDSCs, macrophages, and DCs contribute to immune suppression within the MM marrow through several mechanisms, including STAT3/STAT1-dependent MDSC activation [79], CCL2- and IL-10R/STAT3-associated macrophage programs [80,81], and DC-mediated protection of MM cells from CD8^+^ T-cell killing [82]. Altered lipid metabolism has also been associated with dysfunctional states in NK cells and DCs in MM [83]. Thus, although myeloid and DC dysfunction is well documented in MM, the specific contribution of BMAds to these immune changes remains unclear.

## 5. Bone Marrow Adipocytes and Response to Immunotherapy

**Figure 2:**
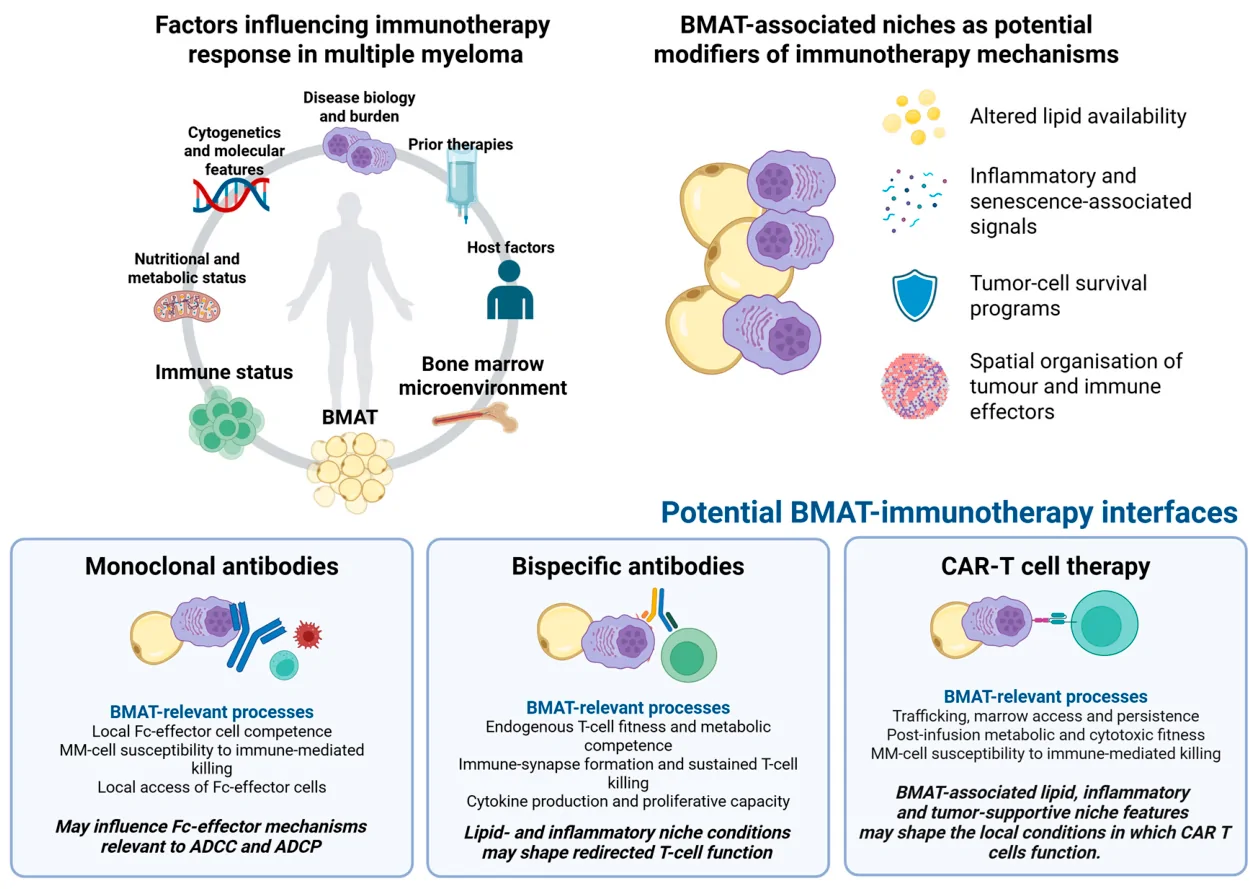
BMAT-related features as potential modifiers of immunotherapy-relevant processes in multiple myeloma. Clinical and marrow factors, BMAT-associated niche features, and potential therapy-specific interfaces are summarized for mAbs, BsAbs, and CAR T-cell therapy. Created with https://BioRender.com/u7jr1yf on 19 August 2026. Abbreviations: ADCC, antibody-dependent cellular cytotoxicity; ADCP, antibody-dependent cellular phagocytosis; BMAT, bone marrow adipose tissue; CAR T cell, chimeric antigen receptor T cell; MM, multiple myeloma.

Current MM immunotherapies include mAbs, ADCs, BsAbs, CAR T cells, and emerging CAR-NK platforms [4,84,85]. Their activity depends on the expression of therapeutic targets and, for several modalities, on the availability and functional competence of endogenous or engineered immune effector cells [86]. It remains unclear whether BMAd-associated metabolic and inflammatory changes affect the processes relevant to immunotherapy within the marrow [87]. Table 2 summarizes the available target- and therapy-specific evidence, and Figure 2 outlines the proposed interfaces between BMAT-associated features and current immunotherapy.

### 5.1. Therapeutic Targets and Ligand-Receptor Axes in BMAds and Adipocyte-Lineage Cells

Direct surface protein evidence for current MM therapeutic targets in primary human BMAds is lacking. For mAbs, ADCs, BsAbs, and CAR-based cellular therapies, a key evidence gap is the cell-type-resolved confirmation of target-surface protein expression on primary human BMAds and adipocyte-lineage cells in MM marrow. The most informative studies would combine surface protein profiling with spatial analysis to determine whether target-positive adipocyte-lineage cells localize near malignant PCs or immune effector cells [98,99]. Primary human BMAds differ from subcutaneous adipocytes and conventional BMSC-derived adipocytes in lipid metabolism and in their inflammatory and secretory profiles [33,34].

Public single-cell BM datasets provide useful information on adipogenic stromal cell populations. However, mature adipocytes are poorly represented in conventional intact-cell single-cell RNA sequencing (scRNA-seq) because their physical properties complicate their isolation [40,100]. The failure to detect a therapeutic target transcript in these datasets does not prove that the corresponding surface protein is absent. Direct cell-type-resolved surface protein assessment of primary human BMAds from MM marrow is still required.

CD38 has been identified as a marker of committed preadipocytes and is implicated in adipose tissue metabolism and inflammation [88,101]. B-cell activating factor (BAFF) and a proliferation-inducing ligand (APRIL), along with related tumor necrosis factor (TNF) superfamily components, have also been reported in adipocytes and adipose tissue models, where they may influence adipogenesis, immune function, and inflammation [91,92]. These observations are relevant because CD38 and BCMA are therapeutic targets in MM, and APRIL-BCMA signaling promotes MM cell survival and growth within the BM microenvironment [93,94]. However, no study identified in this review has established the surface expression of CD38 or BCMA by mature BMAds at levels consistent with clinically relevant bystander targeting or local antigen binding. If CD38 or BCMA is confirmed on BMAds at sufficient and accessible surface densities, several clinical consequences will require evaluation. For anti-CD38 mAbs or BCMA-directed ADCs, target expression on adipocyte-lineage cells could theoretically result in the unintended local engagement of non-malignant cells. BCMA-directed BsAbs and CAR T cells could likewise trigger on-target/off-tumor immune interactions and injury within the adipocytic niche. Conversely, depleting the tumor-supportive adipocyte lineage population may be beneficial. Importantly, adipocyte-derived BAFF or APRIL expression is not equivalent to BCMA surface expression and would not, by itself, establish BMAds as BCMA-expressing bystander cells [91,92]. Future studies should assess CD38 and BCMA using cell-type-resolved surface protein profiling and spatial mapping of primary human BMAds and adipocyte-lineage cells [98,99]. BAFF and APRIL should be examined separately as potential adipocyte-lineage-derived ligands through expression, secretion, and spatial localization studies.

The evidence base for SLAMF7, GPRC5D, and FcRH5 is limited. These molecules are established as PC-associated therapeutic targets in MM [4,84]. We did not identify direct evidence from surface proteins supporting adipocytes as validated antigen-expressing bystander cells for these therapies. Their expression or regulation in BMAds cannot be inferred without direct assessment in BMAd-enriched or adipocyte-lineage compartments from the MM marrow [4,102].

Current evidence points mainly to indirect effects of BMAds on immunotherapy-relevant biology through metabolic and inflammatory niche remodeling, rather than therapeutic-target expression [16,43]. Preliminary spatial data further suggest that BMAT-rich fractions may differ from the surrounding BM in MM cell and immune cell composition, although the functional consequences of this remain unclear [47].

### 5.2. Monoclonal Antibodies

Within the mAb class, anti-CD38 antibodies are particularly relevant to the BMAd–immune axis because their activity depends, in part, on Fc effector mechanisms in the BM microenvironment [89,103]. Daratumumab and isatuximab can also deplete CD38-expressing immunosuppressive cell populations, adding an immune remodeling component that may be influenced by local niche conditions [89,104]. Elotuzumab provides a complementary example of a mAb with a more explicit dependence on NK-cell engagement through FcγRIIIA/CD16-mediated ADCC and SLAMF7-dependent NK-cell co-stimulation [90,105]. Accordingly, mAb activity may be influenced by the local availability and functional competence of NK cells, macrophages, complement, and other Fc-effector components within the BM niche [103].

BMAT-associated immune organization may influence Fc-dependent effector mechanisms, particularly those relevant to elotuzumab and the ADCC component of anti-CD38 therapy. Preliminary paired analyses showed differences in cytotoxic NK cell representation between BMAT-associated fractions and matched aspirates, but did not assess NK cell function, ADCC, or mAb treatment response [47].

Thus, these data do not establish whether BMAT-associated immune organization affects ADCC, ADCP, complement-dependent cytotoxicity, or clinical responses to mAbs, and direct studies linking BMAds to reduced mAb efficacy are lacking.

### 5.3. T-Cell-Redirecting Therapies: CAR T Cells and Bispecific Antibodies

Both CAR T cells and BsAbs depend on T-cell fitness, although BsAbs recruit endogenous T cells, whereas CAR T-cell therapy uses ex vivo engineered T cells [84,106,107]. In MM, pretreatment T-cell characteristics are associated with the response to BsAbs [108,109].

BM lipid exposure impairs CD8^+^ T-cell metabolism and function in MM [41]. Myeloma-exposed BMAds also undergo lipid remodeling [16] and acquire inflammatory and senescence-associated features [43]. The impact of these BMAd-associated changes on redirected T-cell activity during CAR T-cell or BsAb therapy remains unknown. Functional models incorporating BMAds, MM cells, and the relevant T-cell effectors are needed to address this question.

### 5.4. Co-Inhibitory and Target-Independent Niche-Mediated Resistance Pathways

Co-inhibitory and stromal resistance mechanisms may operate alongside BMAd-associated metabolic and inflammatory remodeling in the MM niche. In MM, BMSCs can suppress CD8^+^ T-cell activity through PD-L1/PD-1 signaling [68] and impair NK cell function through CD155/TIGIT signaling [75]. These mechanisms are not BMAd-specific, and mature BMAds have not been shown to contribute directly.

In mice with high-fat diet-induced obesity, BMAd expansion was associated with increased MCP-1 expression in BMAT and a greater abundance of PD-L1^+^ myeloid cells. Preventing BMAd maturation reduced both BMAT MCP-1 expression and PD-L1^+^ myeloid cell abundance [110]; however, this BMAd-associated MCP-1/PD-L1^+^ myeloid axis has not been evaluated in MM.

The stromal protection of MM cells from immune-mediated killing provides an additional target-independent mechanism. BMSCs can reduce MM cell susceptibility to NK and CAR T-cell cytotoxicity through anti-apoptotic programs involving survivin, MCL-1, and XIAP [74,111]. Comparable protection from immune-mediated killing has not been demonstrated for BMAds.

## 6. Host and Treatment Factors Shaping the Myeloma Marrow Niche

Aging, obesity, and prior therapy may further reshape the myeloma marrow niche by altering adipocyte and stromal biology and affecting local immune function, with potential consequences for immunotherapy responses.

### 6.1. Aging-Related Remodeling of the Myeloma Marrow Niche

Aging is associated with increased BM adiposity, stromal cell senescence, and an inflammatory microenvironment [10,11,112]. BM adiposity increases with age, although its distribution and clinical associations vary across skeletal sites [10,12]. In MM, malignant PCs develop within a BM microenvironment shaped by age-related changes in adiposity, stromal function, and immunity [11].

Age-related changes in BMAds may also affect their secretory phenotype. Senescent BMAds have been linked to increased serum amyloid P production and skeletal amyloid deposition in non-MM models [113,114], although the relevance of this pathway in MM remains unknown.

Stromal senescence has also been associated with the progression from MGUS to MM [112], but its consequences for anti-myeloma immunity remain unclear. More directly relevant to immunotherapy, a T-cell-based measure of immune age predicted outcomes with daratumumab-based therapy more accurately than chronological age [115]. A direct link between age-related changes in BMAds and immunotherapy response in MM has not been established.

### 6.2. Obesity-Related Remodeling of the Myeloma Marrow Niche

Obesity can affect MM marrow through systemic inflammation and changes in adipokine and adipocyte biology [116,117,118]. However, body composition does not reliably reflect BMAT because marrow fat varies considerably across skeletal sites [119,120]. In MM models, adipocytes derived from overweight or obese donors increase tumor cell drug resistance [121], whereas evidence directly linking obesity to BMAd-mediated immune dysfunction remains scarce.

In an imaging mass cytometry study of diagnostic BM biopsies from African American patients with MM, morbid obesity was associated with a higher abundance of cytotoxic T cells and shorter distances between cytotoxic T cells and MM cells compared with normal weight individuals [122]. BMAT was not assessed, and responses to mAbs, BsAbs, or CAR-based therapies were not evaluated. Therefore, the observed immune differences cannot be attributed to BMAds or interpreted as evidence of altered immunotherapy efficacy.

Data on obesity and immunotherapy outcomes in patients with MM are also limited. A systematic review and meta-analysis of CAR T-cell studies found no significant association between overweight or obesity and efficacy or toxicity in the MM subgroup, although few data were available [123]. Obesity and BMAT should be considered separately when assessing their potential effects on immunotherapy responses.

### 6.3. Therapy-Related Immune Aging and Fitness for Immunotherapy

Beyond aging and BM adiposity, prior therapy is a major modifier of the myeloma marrow niche, with potential effects on stromal integrity, adipocyte-lineage remodeling, and immune effector fitness. In MM, conventional treatment and ASCT can reshape the T-cell compartment, favoring premature aging phenotypes, mitochondrial dysfunction, and altered fatty acid oxidation [71]. These changes are relevant because they converge on cytotoxic effector cell fitness, a shared requirement for Fc-dependent antibodies, T-cell redirection, and cellular therapy.

Prior therapy may also remodel the adipocyte-lineage niche. This is particularly relevant for glucocorticoids [124], which are frequently included in MM treatment regimens and promote the adipogenic differentiation of BMSCs [125]. In adult mice, glucocorticoid treatment induces rapid senescence in BMAd lineage cells through an oxylipin-PPARγ positive feedback loop [126]. This pathway may converge with myeloma-induced BMAd reprogramming, in which adipocytes acquire metabolic dysfunction, inflammatory activation, and senescence-associated secretory features that support tumor cell fitness and resistance to treatment [43]. No MM study has yet linked glucocorticoid-induced BMAd senescence to T-cell, NK-cell, CAR T-cell, or BsAb-recruited T-cell function.

Pharmacological modulation of PPARγ can modify BM adiposity and adipocyte lineage biology in preclinical models [127,128]. Notably, PPARγ agonists include thiazolidinediones (TZD), insulin-sensitizing drugs used clinically for the treatment of type 2 diabetes [129]. PPARγ-modulating interventions have not been tested as immunotherapy-enhancing strategies in MM and would need to demonstrate attenuation of BMAd-associated lipid stress or inflammatory signaling, along with improved immune effector function.

Treatment-associated changes in the BM microenvironment may persist even after a decrease in tumor burden. Long-term MM survivors can retain BM immune alterations for more than a decade after first-line therapy, including persistent inflammatory immune states, despite durable disease control [130]. In parallel, myeloma-reprogrammed BMAds can sustain bone disease after tumor clearance through EZH2-PPARγ-dependent adipocyte dysfunction, indicating that adipocyte lineage remodeling may persist beyond the apparent tumor response [21]. These observations suggest that reducing the clonal PC burden does not necessarily normalize the stromal, adipocytic, and immune BM microenvironments. Residual immune dysfunction may influence the immune context in which contemporary immunotherapies are used. A T-cell-based metric of immune age has been shown to predict outcomes in older patients receiving daratumumab-based therapy more effectively than chronological age [115]. Similarly, in patients treated with teclistamab, higher pretreatment CD8^+^ effector T-cell populations were associated with response, whereas Treg enrichment was associated with non-response, indicating that the baseline T-cell state can influence the response to T-cell-redirecting therapies [109]. Target expression alone may be insufficient for effective killing when effector-cell fitness is impaired or inhibitory signals are present within the BM niche [41,68,72,109].

Direct studies integrating therapy-related immune aging, aging, obesity, and BMAd/BMAT features within the myeloma marrow are still lacking. Therefore, immunotherapy studies should assess tumor burden and antigen expression alongside BMAd/BMAT features and the functional state of the local immune-stromal compartment.

## 7. Therapeutic Implications and Unresolved Questions

### 7.1. Candidate Pathways for Niche-Directed Intervention

Lipid handling is a potential target for modifying the BMAd-associated niche. MM cells induce adipocyte lipolysis and take up the released fatty acids through FATP1/4 [16]. Lipid uptake is also relevant to CD8^+^ T-cell dysfunction in the MM marrow [41], while FABP4/5 represent additional targets of interest [65]. Future studies should assess whether targeting these pathways improves immune-mediated MM-cell killing, rather than focusing only on effects on MM-cell metabolism.

Leptin signaling provides a more direct link between adipocyte-derived signals and anti-myeloma immunity. Leptin receptor signaling impairs iNKT cell anti-myeloma activity, whereas receptor blockade restores this activity in preclinical models [42]. BMAd senescence may also be a therapeutic target. Senolytic treatment of experimentally senescent adipocytes reduced their protection of MM cells from dexamethasone, supporting senescence as a potential therapeutic target in the adipocytic marrow niche [43]. Its impact on immune-mediated anti-myeloma activity has not yet been examined. No BMAd-directed intervention has yet been shown to improve the activity of mAbs, BsAbs, CAR T cells, or CAR-NK cells in patients with MM.

### 7.2. Combination Strategies and Patient Selection

BMAd-directed interventions have not been tested in combination with immunotherapy for MM. Their value will depend on demonstrating improved immune-mediated killing with mAbs, BsAbs, or CAR T cells, rather than changes limited to BMAT or MM-cell metabolism [16,41,43].

Evidence from non-myeloma settings indicates that BMAT can be pharmacologically modified, with PPARγ-dependent marrow adipogenesis being the best-established axis. Rosiglitazone, a TZD PPARγ agonist, promotes BMAT expansion and impairs stress-induced myelopoiesis. In contrast, data from newer TZD analogs suggest that the adverse skeletal and stromal effects of classical PPARγ agonism may be partially separable from metabolic efficacy [128,131]. In MM, PPARγ activation has shown divergent effects in experimental studies. Earlier in vitro studies have reported that PPARγ ligands induce growth inhibition and apoptosis in MM cells [132,133]. However, pharmacological activation of PPAR has been reported to reduce cereblon expression and attenuate lenalidomide activity in MM models [134]. These divergent findings indicate that the effects of PPAR modulation depend on the compound, PPAR isoform, and treatment context and do not support PPARγ agonism as a strategy to modify BMAT or enhance immunotherapy in MM patients.

Other pathways include adipsin signaling, parathyroid hormone-induced BMAd lipolysis, PPARα-dependent regulation of marrow adiposity, and statin-sensitive adipogenic remodeling, further indicating that BMAT quantity and lipid output can be pharmacologically influenced, although most evidence remains non-MM and model-dependent [135,136,137,138].

Accordingly, these agents are currently more useful as experimental tools for investigating BMAd biology than as immunotherapy-enhancing strategies for MM [128,131,139]. Their value lies in testing whether modulating BMAd lipid handling, inflammatory signaling, or senescence-associated programs can improve immune effector activity in patient-relevant animal models.

The study design should account for treatment timing and the baseline BMAd/BMAT phenotype. Relevant settings include assessments before CAR T-cell infusion or BsAbs initiation, during immune reconstitution, and at relapse. Longitudinal studies should determine whether changes in BMAT burden, BMAd morphology, lipid handling, adipokine secretion, and inflammatory or senescence-associated programs are accompanied by changes in T-cell metabolic fitness, NK cell function, immune synapse formation, tumor cell killing, effector cell persistence, and treatment response.

At baseline, stratification should extend beyond BMI and target antigen expression by integrating BMAd/BMAT measures with body composition, immunosenescence markers, and spatial immune architecture. Such profiling may identify marrow features associated with immunotherapy response, although prospective validation is required [109,115,116,118].

In the NUTRIVENTION trial, greater body mass index (BMI) loss during a high-fiber, plant-based dietary intervention was associated with a trend toward reduced BMAd size in participants with MGUS or SMM and elevated BMI [140]. However, this study did not establish whether these changes directly resulted from BMAd remodeling or affected immune function. The BEAM-MM trial (NCT07564219) is evaluating a ketogenic diet or ketone monoester supplementation in patients receiving BCMA-directed CAR T-cell or BsAb therapy, but BMAT and BMAds are not study endpoints [141]. No clinical study has directly tested the effect of BMAd modification on immunotherapy response in MM.

### 7.3. Validation Priorities for the BMAd-Immune Axis

Despite recent spatial analyses of the MM marrow microenvironment, studies jointly profiling BMAT and immune cell states in situ are lacking. A direct comparison of BMAT-enriched and BMAT-poor regions should determine whether adipocyte-rich areas exhibit distinct immune composition and spatial organization, and whether these features are associated with local metabolic and inflammatory changes [35,98].

Functional validation requires models that incorporate BMAds and cellular components relevant to each immunotherapeutic modality. BsAb models should include antigen-positive MM cells, T cells, and BsAbs, whereas CAR T-cell models should combine antigen-positive MM cells with CAR T cells. For mAbs, the appropriate Fc-effector cells should also be included to capture antibody-dependent cytotoxicity or phagocytic mechanisms. BMAds can then be incorporated as an additional niche component, either directly or through defined BMAd-derived soluble or lipid exposures [39,41]. Relevant endpoints should include MM cell killing, immune synapse formation, cytokine production, T-cell metabolic fitness, and effector cell persistence.

Prospective clinical studies should determine whether BMAd characteristics and their associated immune contexts are linked to treatment responses and clinically relevant outcomes. Such studies should combine longitudinal assessments of the adipocytic niche with immune and metabolic profiling using standardized sampling approaches. RRMM may be a particularly informative setting, given the coexistence of prior treatment exposure, immune dysfunction, and BMAd-associated tumor-supportive mechanisms.

A functional association between BMAds and immunotherapy response should be established before considering BMAd-directed interventions for clinical testing in combination with immune-based therapies.

## 8. Limitations of the Review

This review had several limitations. As a narrative review, it does not provide a systematic or quantitative synthesis of the literature, and selection bias cannot be excluded. The available evidence is heterogeneous and includes studies of primary human BMAds, experimental adipocyte models, non-MM marrow or adipose tissue systems, and preliminary conference data in some areas. Findings from non-MM models provide a biological context but cannot establish BMAd-specific mechanisms in MM. Direct studies linking BMAds to immunotherapy activity remain scarce, and several proposed BMAd-immune interactions still require functional validation.

## 9. Conclusions

BMAds should be considered active components of the myeloma marrow niche rather than a passive histological background. The strongest evidence indicates that myeloma exposure can reprogram these cells toward a metabolically altered and inflammatory phenotype, with potential consequences for tumor metabolism and local immune fitness [16,41,43].

Available evidence does not establish that BMAds act as clinically relevant antigen sinks or bystander targets for MM immunotherapy. BMAd-rich niches may indirectly influence treatment response by shaping the metabolic and inflammatory microenvironment, thereby affecting tumor and immune-cell fitness. However, their functional impact on the efficacy of current MM immunotherapies remains unclear.

Therapeutically, the priority is not to eliminate marrow fat but to identify modifiable BMAd-associated niche programs that could enhance anti-myeloma immune activity. Establishing their clinical relevance will require models and longitudinal studies that resolve adipocyte-immune interactions and directly test their impact on immunotherapy responses.

## Figures and Tables

**Table 1:** BMAd-associated mechanisms with immunometabolic relevance in the multiple myeloma bone marrow niche.

BMAd-Associated Mechanism	Evidence in MM	Immune Relevance and Level of Support	Key Gaps	References
**MM-induced BMAd lipolysis and MM-cell FFA uptake**	In BMAd-MM co-cultures, MM cells promote adipocyte lipolysis, and the released FFAs are transported into MM cells through FATP1/4-dependent mechanisms.	**Potential immunometabolic relevance.** MM-induced BMAd lipolysis may increase local lipid availability; however, no immune cell phenotype or functional endpoints were assessed, and this pathway has not been directly linked to immune dysfunction.	The contribution of MM-induced BMAd lipolysis to immune cell lipid exposure, metabolic fitness, and immune dysfunction in vivo has not yet been established.	[16]
**BM lipid exposure and CD8^+^ T-cell metabolic dysfunction**	Marrow CD8^+^ T cells from patients with MM exhibit increased long-chain FA uptake, reduced mitochondrial mass, and impaired effector functions. Lipid depletion from autologous BM plasma or FATP1 inhibition improves ex vivo CD8^+^ T-cell metabolic fitness and effector function.	**Direct immune evidence.** BM lipid exposure is associated with impaired CD8^+^ T-cell metabolic fitness and effector function.	The cellular origin and composition of lipids available to CD8^+^ T cells, their spatial distribution within MM marrow, and the contribution of BMAd lipolysis to this lipid pool and CD8^+^ T-cell dysfunction remain unknown.	[41]
**Leptin-LEPR signaling**	Adipocyte co-culture and recombinant leptin promote MM-cell proliferation and reduce chemotherapy-induced apoptosis. LEPR blockade attenuates the adipocyte-associated proliferative effect.	**Direct immune evidence**. Leptin impairs iNKT-cell anti-myeloma activity in human and murine MM models, whereas LEPR antagonism enhances iNKT-cell-mediated anti-myeloma activity.	The local marrow concentration and cellular source of leptin in MM, the contribution of BMAd-derived leptin to iNKT cell dysfunction, and the implications of this axis for anti-myeloma immune control remain to be elucidated.	[42,51]
**Adiponectin downregulation and reduction of the IGFBP2^+^ adipocyte-lineage population**	MM-derived TNF-α downregulated adiponectin in the BMAd model. In human MM marrow, the IGFBP2^+^/adiponectin^+^ small adipocyte population is reduced in high-risk disease, and MM co-culture decreases IGFBP2 and adiponectin expression in normal BMSCs. In preclinical models, host-derived adiponectin suppresses MM growth and bone disease.	**Indirect immunometabolic relevance.** Adiponectin is an adipocyte-derived mediator with tumor-suppressive effects in preclinical MM models. Its downregulation may alter the adipokine balance and inflammatory milieu of the MM marrow niche.	The functional and spatial consequences of reduced adiponectin availability and loss of the IGFBP2^+^/adiponectin^+^ adipocyte lineage population on immune cell states, immunoparesis, infection susceptibility, and treatment response remain unclear.	[32,52,53]
**BMAT-associated NK-cell representation**	NK cell frequencies increased in aspirates from patients with MGUS, NDMM, and at follow-up, whereas no corresponding increase was observed in the BMAT-associated compartment, particularly for cytotoxic CD16^high^CD56^low^ NK cells.	**Preliminary immune evidence.** BMAT-associated differences in NK cell representation were most evident within the cytotoxic CD16^high^CD56^low^ NK cell subset.	The phenotype and cytotoxic function of NK cells in BMAT fractions, their spatial localization in BMAT-associated marrow regions, and the relevance of this compartmental pattern to Fc-dependent antibody effector functions remain undefined.	[47]
**FABP4-associated lipid and immune remodeling**	FABP4 expression was increased in BMAds from patients with MM. In high-fat diet MM models, global *Fabp4* deficiency was associated with a lower tumor burden, reduced macrophage infiltration and pro-inflammatory polarization, and altered T helper cell subset representation.	**Preliminary immunometabolic relevance.** FABP4 may link lipid handling to the immune cell composition within the MM marrow niche.	Validation is required to determine whether BMAd-derived FABP4, rather than FABP4 from macrophages, MM cells, or other sources, influences macrophage polarization, T-cell composition, and anti-myeloma immunity.	[54]
**Myeloma-associated BMAd senescence and inflammatory secretory remodeling**	MM-exposed adipocytes acquire metabolic dysfunction and a senescence-associated secretory phenotype, with increased SA-β-galactosidase activity and expression of IL-6, CXCL1, and CXCL2. In co-culture models, dasatinib combined with quercetin attenuated adipocyte-mediated protection against dexamethasone.	**Indirect immunometabolic relevance.** The senescence-associated BMAd secretome may modify the inflammatory and metabolic conditions of the local immune niche.	The effects of senescent BMAds on immune cell metabolism, phenotype, and cytotoxic function, as well as their contribution to immune-mediated MM cell killing, remain undefined.	[43]
**BMAd-induced tumor-cell survival programs**	Mature BMAds induce autophagy-related programs and reduce chemotherapy-induced apoptosis in MM cells. Exosomes from MM-associated adipocytes can also transfer pro-survival lncRNAs, including LOC606724 and SNHG1, thereby attenuating bortezomib-induced apoptosis in MM cells.	**Potential immune relevance.** BMAd-induced tumor-cell survival programs may influence MM cell susceptibility to cytotoxic effector mechanisms within the marrow niche.	The effects of BMAd-induced autophagy and extracellular vesicle transfer on MM cell susceptibility to immune-mediated killing remain undetermined.	[15,55]

Abbreviations: BM, bone marrow; BMAd, bone marrow adipocyte; BMAT, bone marrow adipose tissue; BMSC, bone marrow stromal cell; CXCL1, C-X-C motif chemokine ligand 1; CXCL2, C-X-C motif chemokine ligand 2; FA, fatty acid; FABP4, fatty acid-binding protein 4; FATP1/4, fatty acid transport proteins 1 and 4; Fc, fragment crystallizable; FFA, free fatty acid; IGFBP2, insulin-like growth factor-binding protein 2; IL-6, interleukin 6; iNKT, invariant natural killer T cell; LEPR, leptin receptor; lncRNA, long noncoding RNA; MGUS, monoclonal gammopathy of undetermined significance; MM, multiple myeloma; NDMM, newly diagnosed multiple myeloma; NK, natural killer; SA-β-galactosidase, senescence-associated β-galactosidase; TNF-α, tumor necrosis factor alpha; Bold text indicates the level of immune evidence.

**Table 2:** Therapeutic targets and immunotherapy modalities in multiple myeloma: relevance of BMAd- and BMAT-associated biology.

Therapeutic Target	Therapeutic Modalities and Representative Agents	Available Evidence for Target Expression in BMAds or Adipocyte-Lineage Cells	BMAd/BMAT-Associated Evidence Relevant to Therapeutic Activity	References
**CD38**	**mAbs:** daratumumab; isatuximab	CD38 has been reported in committed preadipocytes in the non-marrow adipose tissue. Direct cell-surface evidence for CD38 expression in mature primary human BMAds or adipocyte-lineage cells in MM marrow has not been identified.	Anti-CD38 mAbs can engage NK cell-mediated ADCC and macrophage-mediated ADCP, among other mechanisms of action. Preliminary paired BMAT–aspirate analyses showed that the increase in cytotoxic NK cell representation observed in aspirates was not detected in the corresponding BMAT-associated fractions. Its relevance to ADCC, ADCP, complement-dependent cytotoxicity, and anti-CD38 treatment response remains untested.	[47,88,89]
**SLAMF7**	**mAb:** elotuzumab	Direct cell-surface evidence for SLAMF7 expression in mature primary human BMAds or adipocyte-lineage cells has not yet been identified.	Elotuzumab activates NK cells through CD16-dependent and CD16-independent mechanisms, with CD16-mediated ADCC representing an important Fc-effector component. Therefore, the preliminary BMAT-associated differences in cytotoxic NK cell representation may be relevant; however, NK cell function and elotuzumab-mediated cytotoxicity have not been assessed in BMAd-containing MM models.	[47,90]
**BCMA**	**ADC:** belantamab mafodotin **BsAbs:** teclistamab; elranatamab, linvoseltamab **CAR T cells:** idecabtagene vicleucel; ciltacabtagene autoleucel **CAR-NK cells:** investigational BCMA-directed platforms	Direct evidence for surface BCMA expression on mature primary human BMAds or adipocyte-lineage cells in MM marrow is lacking. In non-marrow adipocytes and adipose tissue models, BAFF expression increases during adipocyte differentiation, and BCMA, BAFF-R, and TACI have been detected in adipocyte-lineage cells. BAFF and APRIL have also been described in adipose tissue models. In MM, BAFF and APRIL support MM cell survival, whereas APRIL-BCMA signaling promotes MM cell proliferation, survival, and immunosuppression in the BM microenvironment. These findings do not establish a functional BMAd-BAFF/APRIL-BCMA axis in MM.	**ADC.** Mature BMAds induce autophagy-related programs and reduce chemotherapy-induced apoptosis in MM cells. Exosomes from MM-associated adipocytes transfer pro-survival lncRNAs and attenuate bortezomib-induced apoptosis. These mechanisms have not been assessed with BCMA-directed ADCs. **BsAbs and CAR T cells.** BM lipid exposure impairs CD8^+^ T-cell metabolic fitness and effector functions. MM-induced BMAd lipolysis occurs in separate co-cultures, but its contribution to the lipid pool affecting T cells remains unproven. We did not identify any studies that assessed the effects of BMAd on BsAb- or CAR T-cell activity. **CAR-NK cells.** Available BMAT-associated data describe NK cell representation but do not assess conventional NK cell function or CAR-NK cell activity.	[15,16,41,47,55,91,92,93,94]
**GPRC5D**	**BsAb:** talquetamab, forimtamig **CAR T cells:** MCARH109 and other investigational products	Direct cell-surface evidence for GPRC5D expression in mature primary human BMAds or adipocyte-lineage cells has not yet been identified.	No GPRC5D-specific BMAd/BMAT data were identified, and BMAd effects have not been assessed in GPRC5D-directed BsAbs or CAR T-cell models.	[95,96]
**FcRH5**	**BsAb:** cevostamab	Direct cell-surface evidence for FcRH5 expression in mature primary human BMAds or adipocyte-lineage cells has not yet been identified.	No FcRH5-specific BMAd/BMAT data were identified, and BMAd effects have not been assessed with FcRH5-directed T-cell redirection.	[97]

Abbreviations: ADCC, antibody-dependent cellular cytotoxicity; ADCP, antibody-dependent cellular phagocytosis; ADC, antibody–drug conjugate; APRIL, a proliferation-inducing ligand; BAFF, B-cell activating factor; BCMA, B-cell maturation antigen; BMAd, bone marrow adipocyte; BMAT, bone marrow adipose tissue; BsAb, bispecific antibody; CAR, chimeric antigen receptor; FcRH5, Fc receptor homolog 5; GPRC5D, G protein-coupled receptor class C group 5 member D; lncRNA, long non-coding RNA; mAb, monoclonal antibody; MM, multiple myeloma; NK, natural killer; SLAMF7, signaling lymphocytic activation molecule family member 7. Bold text indicates therapeutic targets and therapeutic modality categories.

## Data Availability

No new data were created or analyzed during this study. Data sharing does not apply to this article.

## Abbreviations

The following abbreviations are used in this manuscript:

ABC	ATP-binding cassette
ADC	Antibody-drug conjugate
ADCC	Antibody-dependent cellular cytotoxicity
ADCP	Antibody-dependent cellular phagocytosis
AhR	Aryl hydrocarbon receptor
AI	Artificial intelligence
AKT	Protein kinase B
APRIL	A proliferation-inducing ligand
ASCT	Autologous stem-cell transplantation
ATG3	Autophagy-related 3
ATG5	Autophagy-related 5
ATP	Adenosine triphosphate
BAFF	B-cell activating factor
BCMA	B-cell maturation antigen
BM	Bone marrow
BMAd	Bone marrow adipocyte
BMAT	Bone marrow adipose tissue
BMI	Body mass index
BMSC	Bone marrow stromal cell
BsAb	Bispecific antibody
CAR T cell	Chimeric antigen receptor T cell
CAR-NK	Chimeric antigen receptor natural killer cell
cBMAT	Constitutive bone marrow adipose tissue
CCL2	C-C motif chemokine ligand 2
CCRL2	C-C motif chemokine receptor-like 2
CDC	Complement-dependent cytotoxicity
CMKLR1	Chemerin chemokine-like receptor 1
CSE-PDFF	Chemical shift-encoded proton-density fat fraction
CTLA-4	Cytotoxic T-lymphocyte-associated protein 4
CXCL1	C-X-C motif chemokine ligand 1
CXCL12	C-X-C motif chemokine ligand 12
CXCR4	C-X-C chemokine receptor type 4
DC	Dendritic cell
DNMT3A	DNA methyltransferase 3 alpha
DPP4	Dipeptidyl peptidase-4 (CD26)
ERK	Extracellular signal-regulated kinase
EZH2	Enhancer of zeste homolog 2
FABP5	Fatty acid-binding protein 5
FAPα	Fibroblast activation protein alpha
FATP	Fatty acid transport protein
FATP1	Fatty acid transport protein 1
FcRH5	Fc receptor homolog 5
FA	Fatty acid
FABP4	Fatty acid-binding protein 4
FATP4	Fatty acid transport protein 4
FF	Fat fraction
FFA	Free fatty acid
GFP	Green fluorescent protein
GPRC5D	G protein-coupled receptor, class C, group 5, member D
GREM1	Gremlin 1
ICAM1	Intercellular adhesion molecule 1
IGF-1	Insulin-like growth factor 1
IGFBP2	Insulin-like growth factor-binding protein 2
IL-10	Interleukin 10
IL-6	Interleukin 6
ILC	Innate lymphoid cells
iNKT	Invariant natural killer T cell
ISS	International Staging System
JAK	Janus kinase
LC3	Microtubule-associated protein 1 light chain 3
LEPR	Leptin receptor
lncRNAs	Long noncoding RNAs
MCL-1	Myeloid cell leukemia 1
MCP-1	Monocyte chemoattractant protein 1
MDSC	Myeloid-derived suppressor cell
MGUS	Monoclonal gammopathy of undetermined significance
MHC	Major histocompatibility complex
MM	Multiple myeloma
MM-BMSCs	Bone marrow stromal cells from patients with multiple myeloma
MRI	Magnetic resonance imaging
NDMM	Newly diagnosed multiple myeloma
NF-κB	Nuclear factor kappa B
NK	Natural killer cell
NKT	Natural killer T cell
PC	Plasma cell
PD-1	Programmed cell death protein 1
PD-L1	Programmed death-ligand 1
PI3K	Phosphoinositide 3-kinase
PPARα	Peroxisome proliferator-activated receptor alpha
PPARγ	Peroxisome proliferator-activated receptor gamma
PUFA	Polyunsaturated fatty acid
R-ISS	Revised International Staging System
rBMAT	Regulated bone marrow adipose tissue
RRMM	Relapsed/refractory multiple myeloma
SASP	Senescence-associated secretory phenotype
scRNA-seq	Single-cell RNA sequencing
SDF-1α	Stromal cell-derived factor 1 alpha
SERPINB2	Serpin family B member 2
sFF	Signal fat fraction
SLAMF7	Signaling lymphocytic activation molecule family member 7
SMM	Smoldering multiple myeloma
STAT3	Signal transducer and activator of transcription 3
TGF-β	Transforming growth factor beta
Th17	T helper 17 cell
TIGIT	T cell immunoreceptor with Ig and ITIM domains
TLR9	Toll-like receptor 9
TNF-α	Tumor necrosis factor alpha
Treg	Regulatory T cell
TZD	Thiazolidinedione
VEGFA	Vascular endothelial growth factor A
XIAP	X-linked inhibitor of apoptosis protein
